# Heat-induced-radiolabeling and click chemistry: A powerful combination for generating multifunctional nanomaterials

**DOI:** 10.1371/journal.pone.0172722

**Published:** 2017-02-22

**Authors:** Hushan Yuan, Moses Q. Wilks, Georges El Fakhri, Marc D. Normandin, Charalambos Kaittanis, Lee Josephson

**Affiliations:** Gordon Center for Medical Imaging, Massachusetts General Hospital, Harvard Medical School, 149, 13^th^ Street, Charlestown, Massachusetts, United States of America; The Chinese University of Hong Kong, HONG KONG

## Abstract

A key advantage of nanomaterials for biomedical applications is their ability to feature multiple small reporter groups (multimodality), or combinations of reporter groups and therapeutic agents (multifunctionality), while being targeted to cell surface receptors. Here a facile combination of techniques for the syntheses of multimodal, targeted nanoparticles (NPs) is presented, whereby heat-induced-radiolabeling (HIR) labels NPs with radiometals and so-called click chemistry is used to attach bioactive groups to the NP surface. Click-reactive alkyne or azide groups were first attached to the nonradioactive clinical Feraheme (FH) NPs. Resulting “Alkyne-FH” and “Azide-FH” intermediates, like the parent NP, tolerated ^89^Zr labeling by the HIR method previously described. Subsequently, biomolecules were quickly conjugated to the radioactive NPs by either copper-catalyzed or copper-free click reactions with high efficiency. Synthesis of the Alkyne-FH or Azide-FH intermediates, followed by HIR and then by click reactions for biomolecule attachment, provides a simple and potentially general path for the synthesis of multimodal, multifunctional, and targeted NPs for biomedical applications.

## Introduction

The value of multimodal (detectable more than one modality) or multifunctional (therapeutic and diagnostic) nanomaterials has been discussed[1] and been the subject of recent comprehensive reviews.[2, 3] Here we provide a facile and general approach to the design of targeted, theranostic nanoparticles (NPs) whereby Feraheme (FH) nanoparticles (NPs) undergo a heat induced radiolabeling (HIR) for detection by nuclear techniques, followed by biomolecule attachment to the NP surface using mild click chemistry reactions. The HIR technique employs heat (120°C, 2 hr) to bind cations to the surface of 5 nm iron oxides.[4, 5]

Though one of several chelator-free methods for radiolabeling nanomaterials,[6] HIR is unique in its ability to label a NP drug with any of three cations used in PET or SPECT (^64^Cu^2+^, ^111^In^3+^ or ^89^Zr^4+^).[4, 5] These procedures yield MRI detectable FH NPs that can be labeled with therapeutic radiometals like ^111^In^3+^ (a low dose SPECT isotope and high dose therapeutic [7]) or ^67^Cu^2+^ [8]. In addition, FH (1) has been shown to bind a wide range of non-radioactive metals by ICP-MS [4]. Given the broad availability of Feraheme (FH) NPs, the radiocation flexibility of the HIR reaction, and the many commercially available click reagents, we reasoned that HIR and click chemistry surface functionalization might make a useful and general combination for the synthesis of multimodal (radioactive, magnetic, fluorescent), targeted NPs if the azide and alkyne groups survived the HIR reaction.

**Fig 1:**
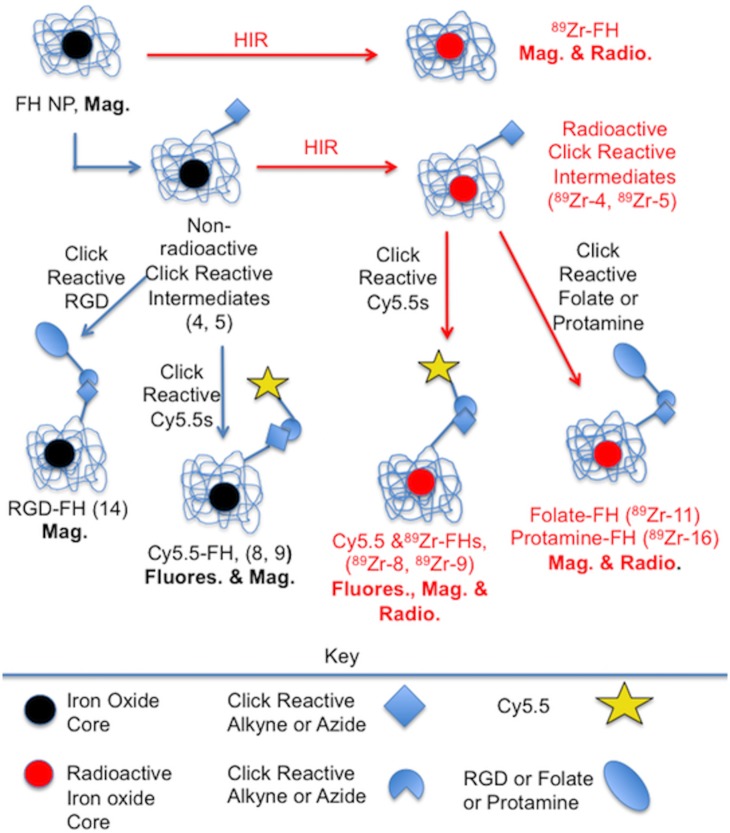
Outline of heat induced radiolabeling (HIR) and click chemistry surface functionalization used to obtain multimodal, targeted NPs. Our previous HIR of FH NPs (top) yielded non-surface functionalized, radioactive NPs (red core). The alkyne and azide functionalized FH intermediates were synthesized (Azide-FH, **4** and Alkyne-FH, **5**) and labeled by HIR reaction, to yield ^89^Zr-Azide-FH (^**89**^**Zr-4**) and ^89^Zr-Alkyne-FH (^**89**^**Zr-5**). Imaging detection modalities for the NPs are in bold. NPs targeted to folate receptors (^89^Zr-Folate-FH, ^**89**^**Zr-11**), integrins (RGD-FH, **14**) or NPs with protamines (^89^Zr-Cy5.5-Protamine-FH, ^**89**^**Zr-16**) were then synthesized. Detailed synthetic schemes are given in Figs 2–4.

**Fig 2:**
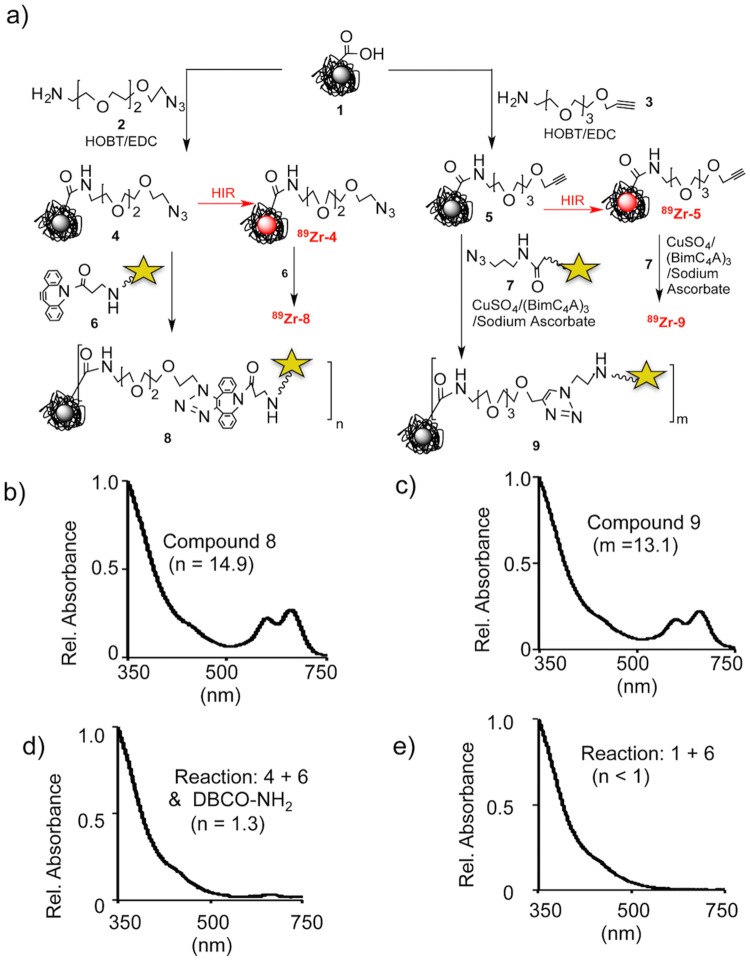
Surface functionalization of Feraheme (FH, 1) with azide or alkyne groups and radiolabeling functionalized NPs by HIR. a) Using FH (**1**)**,** the Azide-FH (**4**) and Alkyne-FH (**5**) were synthesized. Portions of Azide-FH **(4)** and Alkyne-FH (**5**) were then radiolabeled by HIR, yielding ^89^Zr-Azide-FH (^**89**^**Zr-4**) and ^89^Zr-Alkyne-FH (^**89**^**Zr-5**). To determine reactive azide or reactive alkynes, NPs were reacted with the appropriate click reactive Cy5.5 fluorochromes, with Cy5.5s shown as the yellow stars of Fig 1. After removal of the unreacted Cy5.5s (DBCO-Cy5.5, **6** or Azide-Cy5.5, **7**), the number of Cy5.5’s per NP was determined from absorption spectra examples of which are shown in Fig 2b–2e. Controls for covalent binding were a reaction of FH (**1**) and DBCO-Cy5.5 (**6**) and a reaction of Azide-FH **(4)** and DBCO-Cy5.5 (**6**) preoccupied with DBCO-NH_2_. Values in parentheses are the numbers of reactive groups per NP with values summarized in Table 1.

**Fig 3:**
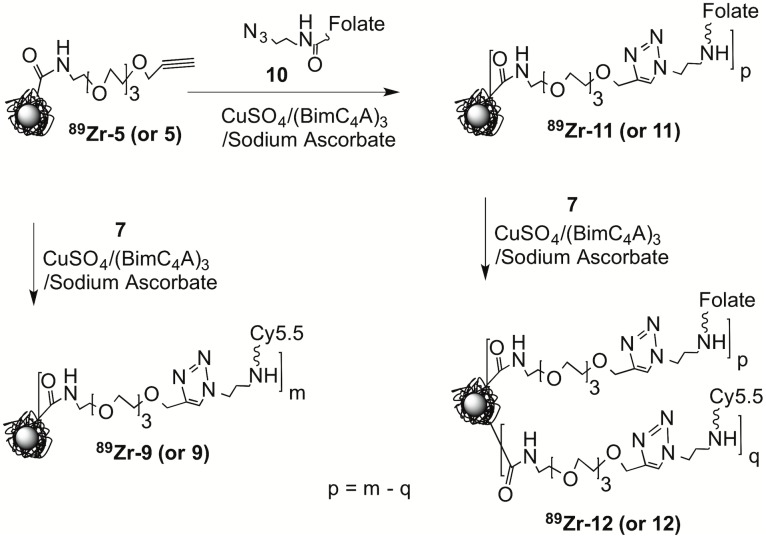
Synthesis of HIR NPs functionalized with folate and determination of folates per NP by the “Before and After Fluorochrome Reaction and Subtraction Method.” Non-radioactive Alkyne-FH (**5**) or radioactive Alkyne-FH (^89^Zr-Alkyne-FH, ^**89**^**Zr-5**) was reacted with the Azide-Cy5.5 (**7)** before reaction with an azide bearing folate **(10).** After reaction with Azide-Cy5.5 (**7**), the number of folate groups, p, on the NP was determined as reactive alkynes before and after folate reaction; that is as ^89^Zr-Cy5.5-Alkyne-FH (^**89**^**Zr-9**) (m) minus the number on ^89^Zr-Cy5.5-Folate-FH (^**89**^**Zr-12**) (q).

**Fig 4:**
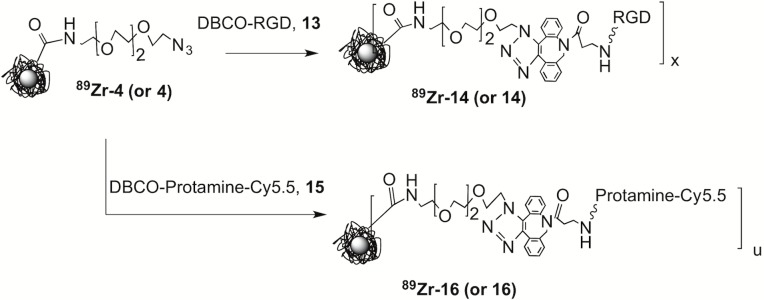
Synthesis of RGD and protamine surface functionalized NPs. ^89^Zr-Azide-FH (^**89**^**Zr-4**) or Azide-FH (**4**) was reacted with DBCO-PEG_4_-RGD (**13**) and the number of RGDs per NP on ^89^Zr-RGD-FH (^**89**^**Zr-14**) or RGD-FH (**14**) was determined by the *“Before and After Fluorochrome Reaction and Subtraction Method”*. DBCO-Protamine-Cy5.5 (**15**) has a C-terminal Cy5.5 was synthesized (see S2 File), and this compound was used to synthesize ^89^Zr-Cy5.5-Protamine-FH (^**89**^**Zr-16**) or Cy5.5-Protamine-FH (**16).**

We therefore combined HIR and click chemistry surface functionalization as summarized in Fig 1 and provided in detail in Figs 2–4. The starting FH NP (Fig 1, upper left) has a 5 nm core of superparamagnetic iron oxide (black central ball) consisting of approximately 5874 Fe/NP (manufacturer’s package insert). Surrounding the core is a thick (10 nm) coating of polymeric carboxymethyl dextran (CMD, light blue lines), so the NP has an overall size of 20–25 nm.[9] Previously, this starting FH NP was subjected to the HIR reaction, shown here as the creation of a radioactive FH (red ball in figures).[4, 5] Here we used FH NPs for the synthesis of two non-radioactive click reactive intermediates; these were the azide functionalized NP (Azide-FH, **4**) or an alkyne functionalized NP (Alkyne-FH, **5**). The NPs, Azide-FH **(4)** and Alkyne-FH, (**5**), were then reacted with commercially available, click reactive Cy5.5 fluorochromes, to demonstrate the presence reactive azides and alkynes on the NP surface and provide a path to nonradioactive but magnetic and fluorescent NPs for future studies. Finally, Azide-FH **(4)** and Alkyne-FH (**5**) were subjected to HIR; our demonstration uses ^89^Zr and the number of reactive azides and alkynes on the resulting ^89^Zr-Azide-FH, (^**89**^**Zr-4**) and ^89^Zr-Alkyne-FH (^**89**^**Zr-5**) NPs were compared with the number on the precursor (Azide-FH, **4** and Alkyne-FH, **5**) NPs to determine whether azide and alkyne groups survived the HIR reaction. Since the alkynes and azides survived HIR (see below and Table 1), the ^89^Zr-Azide-FH (^**89**^**Zr-4**) and ^89^Zr-Alkyne-FH (^**89**^**Zr-5**) NPs were then reacted with click reactive targeting molecules, yielding ^89^Zr-Folate-FH (^**89**^**Zr-11**), ^89^Zr-RGD-FH (^**89**^**Zr-14),** and ^89^Zr-Cy5.5-Protamine-FH (^**89**^**Zr-16**) as shown in Figs 3–4.

**Table 1:** Summary of HIR NP’s and Surface Functionalization Reactions.

Fig	NPs	RG/NP^[^^a^^]^ TG/NP^[^^a^^]^	Diam. ^[^^b^^]^	R_1_^[^^c^^]^ *& (R*_*2*_*)*^[^^c^^]^	Detection Modalities (D) & Targeting (T)^[^^d^^]^
2	FH (**1**)		19.05±0.34	30.10±0.62 *(65*.*35±4*.*31)*	D: **M &** T: N/A
2	Azide-FH (**4**)		19.82±0.43	33.90±1.11 *(68*.*64±1*.*89)*	D: **M &** T: N/A
2	Alkyne-FH (**5**)		20.42±0.26	32.63±0.02 *(68*.*15±0*.*37)*	D: **M** & T: N/A
2	Cy5.5-Azide-FH (**8**)	14.9 Azides	^[^^b^^]^	34.23±1.34 *(65*.*50±3*.*20)*	D: **M, F** & T: N/A
2	Cy5.5-Alkyne-FH (**9**)	13.1 Alkynes	^[^^b^^]^	32.57±1.80 (*67*.*60±0*.*21)*	D: **M, F** & T: N/A
2	^89^Zr-Cy5.5-Azide-FH (^**89**^**Zr-8**)	14.5 Azides			D: **M, F, R &** T: N/A
2	^89^Zr-Cy5.5-Alkyne-FH (^**89**^**Zr-9**)	12.2 Alkynes			D: **M, F, R** & T: N/A
3	Folate-FH (**11**)	10.5 Folates	27.70±2.46	29.50±0.46 *(76*.*88±3*.*77)*	D: **M** & T: **Folate**
3	^89^Zr-Folate-FH (^**89**^**Zr-11**)	12.2 Folates			D: **M, R &** T: **Folate**
4	RGD-FH (**14**)	11.7 RGDs	23.18±0.94	33.05±4.00 *(66*.*74±8*.*44)*	D: **M** & T: **RGD**
4	^89^Zr-RGD-FH (^**89**^**Zr-14**)	14.1 RGDs			D: **M, R** & T: **RGD**
4	Cy5.5-Protamine-FH (**16**)	3.4 Protamines	^[^^b^^]^	22.87±2.01 *(94*.*53±3*.*72)*	D: **M** & T: **Protamine**
4	^89^Zr-Cy5.5-Protamine-FH (^**89**^**Zr-16**)	2.6 Protamines			D: **M. R** & T: **Protamine**

^a^ **RG** = reactive groups which can be azides or alkynes; **TG** = targeting groups which can be RGD or folate or protamine;

^b^ **Diam.** = diameters by light scattering in nm; the sizes of compounds Cy5.5-Azide-FH (**8**), Cy5.5-Alkyne-FH (**9**), and Cy5.5-Protamine-FH (**16**) were not measured because of the interference from Cy5.5.

^c^ **R1** & **R2** = relaxivities at 0.47 T in units of (sec mM)^-1^;

^d^ **M** = magnetic related detections including MRI; **F** = fluorescence imaging; **R** = radioactivity related detections; **Folate** = folate receptor targeting; **RGD** = RGD/integrin mediated targeting; **Protamine** = protamine’s cell membrane translocation; **N/A** = not applicable due to absent function or synthetic intermediate status.

## Materials and methods

### General information

All water was Chelex treated. Reactivial was from Wheaton (0.3 ml, W986253NG). Chemicals/vendors were Feraheme/AMAG Pharmaceuticals; ^89^Zr oxalate/Perkin Elmer @ 0.15GBq/nmol in 1M oxalate; desferoxamine/Sigma (D9533); Azido-PEG_3_-Amine (**2**), Alkyne-PEG_4_-Amine (**3**), DBCO-Cy5.5 (**6**), DBCO-PEG_4_-NHS e**s**ter (A102P-10), and Azide-Cy5.5 (**7**)/all were from Click Chemistry Tools; Azide-Folate (**10**)/Barry & Associates; (BimC_4_A)_3_ (696854-250MG, tripotassium 5,5′,5′′-[2,2′,2′′-nitrilotris(methylene)tris(1*H*-benzimidazole-2,1-diyl)]tripentanoate hydrate)/Sigma-Aldrich. HOBT/#H0468-TCI; EDC/ #22980-Thermo Scientific; Protamine/APP Pharmaceuticals; Cy5.5-amine/470C0-Lumiprobe. All the other solvents and chemicals were from Sigma-Aldrich. Elution profiles from PD-10 columns (GE Healthcare) were analyzed with a Perkin Elmer Wizard 2480 Gamma Counter. DBCO-PEG_4_-RGD (**13,** Fig 4) was synthesized as described [10]. Relaxation times were determined on a Bruker Mini SPEC MQ20, and particle sizes determined on a Malvern Instruments, ZetaSizer Nano Series, Nano-ZS. UV spectra were on Thermo Scientific, Evolution 300 UV-VIS. NPs were washed and concentrated by ultrafiltration (50 kDa Amicon ultrafilters and centrifugation). HPLC (Varian ProStar detector and delivery modules) employed an eluant A (0.1% TFA /water) and eluant B (0.1% TFA in 9.9% water in acetonitrile). Iron was quantified by absorbance at 450 nm using dilutions of 30 mg Fe/ mL Feraheme stock drug for a standard curve.

### Syntheses of Azide-FH (4) and Alkyne-FH (5), see Fig 2a

FH (**1**, 400 μL, 12 mg Fe) was transferred to 0.1 M, pH 6.0–6.4 MES by use of a PD-10 column equilibrated with this buffer. For Azide-FH (**4)**, FH (**1**, 12 mg Fe in 2.1 mL MES buffer), HOBT (10.55 mg, 0.078 mmol), and EDC (55.27 mg, 0.288 mmol) were incubated (room temperature, 20 min). A DMSO solution of Azido-PEG_3_-Amine (**2**, 1 M, 80 μL) was added (50°C, 2 h). Low molecular weight materials were removed and NP concentrated by ultrafiltration. The NP was by PD-10 (chelex treated water) purified and concentrated by ultrafiltration to obtain a stock solution (5mg Fe/mL in chelex water (2.2 mL)) for the copperless click reaction. For Alkyne-FH **(5)**, a solution of Alkyne-PEG_4_-Amine in DMSO (**3,** 0.1 M, 800 μL) was reacted with FH (**1)**, purified and concentrated as for Azide-FH **(4)** and also obtained as a 5 mg Fe/mL in chelex water.

### Demonstration of covalent click reaction, see Fig 2

The azide groups on the Azide-FH NP (**4)** (0.32 mg Fe in water 200 μL) was blocked by addition of DBCO-amine (Click Chemistry Tools, A103, 3.2 μL, 20 mM in DMSO) in a 0.3 mL glass vial (room temperature, 1 h). NPs were DPBS PD-10 purified and concentrated by ultrafiltration. DPBS (200 μL) and DBCO-Cy5.5 (**6**, 6.85 μL, 6.78 mM in DMSO) were added to the retentate and incubated (room temperature, 1 hr). NPs were PD-10 purified, concentrated by ultrafiltration and the number of Cy5.5/NP (1.3) determined by absorbance (Fig 2d). A second control was FH (**1**) (0.32 mg Fe) in chelexed water (200 μL) reacted with DBCO-Cy5.5 (**6**, 6.85 μL, 6.78 mM in DMSO, room temperature, 1 h). The NP was purified and Cy5.5/NP (<1) determined as above (Fig 2e).

### Determination of azides on Azide-FH (4) by reaction with DBCO-Cy5.5 (6), Fig 2

Azide-FH (**4**, 40 μL, 0.2 mg Fe), DBCO-Cy5.5 (**6**, 6.85 μL, 6.78 mM in DMSO), and DPBS (160 μL) were incubated (room temperature, 1 h) followed by a PD-10 (in PBS) purification. The NP Cy5.5-Azide-FH (**8)** was concentrated by ultrafiltration.

### Determination of alkynes on Alkyne-FH (5) by reaction with Azide-Cy5.5 (7)

Alkyne-FH (**5**) (40 μL, 0.2 mg Fe), Azide-Cy5.5 (**7**, 11 μL, 4.225 mM in DMSO), and Cu-(BimC_4_A)_3_-ascorbate catalyst stock solution (5 μL, see S1 File) and DPBS (160 μL) were incubated at room temperature for 15 min followed by a PD-10 (in PBS) purification. The NP Cy5.5-Alkyne-FH **(9)** was concentrated by ultrafiltration.

### Syntheses of ^89^Zr-Azide-FH (^89^Zr-4) and ^89^Zr-Alkyne-FH (^89^Zr-5), see Fig 2a

In a 0.3 mL glass reactivial with magnetic stir bar was placed ^89^Zr-oxalate (0.744 mCi, 1 M oxalic acid, 42 μL) with chelex water (50 μL) and the mixture was brought to pH 8–9 with 1M Na_2_CO_3_. Azide-FH (**4**, 120 μL, 0.6 mg Fe) was added and pH was adjusted. The mixture was heated (120°C, 2 hr, silicon oil bath) and then cooled (ice bath, 15 min, stirring). DFO (3 μL, 20 mM, pH 7.5) was added to remove loosely bound ^89^Zr (15 min). The ^89^Zr-Azide-FH (^**89**^**Zr-4**) was purified by PD-10 (PBS) and concentrated by ultrafiltration, yielding ^89^Zr-Azide-FH (^**89**^**Zr-4**) (1.3 mg Fe/ml) in DPBS (pH 7.4) for copperless click reactions. The product was analyzed for purity by PD-10 in PBS (RCP > 99%, S1 Fig). RCY: 0.672 mCi, 90%. Specific activity: 62.8 mCi/mmol Fe. Synthesis of ^89^Zr-Alkyne-FH (^**89**^**Zr-5**) was as for ^89^Zr-Azide-FH (^**89**^**Zr-4**), yielding ^89^Zr-Alkyne-FH (^**89**^**Zr-5**) (1.3 mg Fe/ml) in DPBS (pH 7.4) for copper mediated click reactions. ^89^Zr-Alkyne-FH (^**89**^**Zr-5**) was analyzed by PD-10 (RCP >99%, S2 Fig). RCY: 0.527 mCi, 87.8%; Specific activity: 49.3 mCi/mmol Fe.

### Reaction of ^89^Zr-Azide-FH (^89^Zr-4) and ^89^Zr-Alkyne-FH (^89^Zr-5) with Cy5.5s, Fig 2

To a 0.3 mL glass reactivial with magnetic stir bar were mixed ^89^Zr-Azide-FH (^**89**^**Zr-4**) (0.2 mg Fe, 150μl, 0.208 mCi) and DBCO-Cy5.5 (**6**, 6.85 μL, 6.78 mM in DMSO). The mixture was incubated (room temperature, 1 hr) with ^89^Zr-Cy5.5-Azide-FH (^**89**^**Zr-8**) obtained by PD-10 purification and ultrafiltration. ^89^Zr-Cy5.5-Azide-FH (^**89**^**Zr-8**) was analyzed by PD-10 gel-filtration (S3 Fig) and formulated in DPBS (pH 7.4) (200 μL). RCY: 0.17 mCi, 81.7%; Specific activity: 47.6 mCi/mmol Fe; RCP >99% (S3 Fig). The ratio of Cy5.5/FHNP (14.5) (Table 1) indicated reactive azides on the starting ^89^Zr-Azide-FH (^**89**^**Zr-4**). Similarly, ^89^Zr-Alkyne-FH (^**89**^**Zr-5**) (0.16 mg Fe, 117 μL, 0.135 mCi), Azide-Cy5.5 (**7**, 11 μL, 4.225 mM in DMSO), and Cu-(BimC4A)_3_-Ascorbate stock solution (5 μL, see S1 File) was incubated under magnetic stirring (room temperature, 15 min). ^89^Zr-Cy5.5-Alkyne-FH (^**89**^**Zr-9**) was purified, collected, and concentrated as for ^89^Zr-Cy5.5-Azide-FH (^**89**^**Zr-8**). The RCP was analyzed by PD-10 (RCP >99%, S4 Fig). RCY: 0.111 mCi, 82.2%. Specific activity: 38.9 mCi/mmol Fe. The ratio of Cy5.5/FHNP (12.2) (Table 1).

### Syntheses of the folate functionalized FHs (Folate-FH, 11, ^89^Zr-Folate-FH, ^89^Zr-11, ^89^Zr-Cy5.5-Folate-FH, ^89^Zr-12), see Fig 3

Alkyne-FH (**5,** 0.2 mg Fe), Azide-Folate (**10**, 4.6 μL, 10 mM in DMSO), a Cu-(BimC_4_A)_3_-Ascorbate stock solution (5 μL, see S1 File) and 200 μL DPBS were incubated (room temperature, 15 min.) Folate-FH (**11)** was purified, collected, and concentrated as for ^89^Zr-Cy5.5-Azide-FH (^**89**^**Zr-8**). The ratio of folate/FHNP (10.5, Table 1) was calculated by determining the numbers of Cy5.5/NP before and after reaction of Azide-Folate (**10**) using Azide-Cy5.5 (**7**)**.** ^89^Zr-Folate-FH (^**89**^**Zr-11**) was synthesized as for Folate-FH (**11**) but with ^89^Zr-Alkyne-FH (**5**). RCY: 0.111 mCi, 64.2%; Specific activity: 30.9 mCi/mmol Fe; RCP >99%. See S5 Fig. Ratio of folate/FHNP was 12.2, (Table 1). ^89^Zr-Folate-FH (^**89**^**Zr-11**) (0.2mg Fe, 150 μL DPBS, 0.173 mCi) with Azide-Cy5.5 (**7**, 11 μL, 4.225 mM in DMSO) by incubation at room temperature for 15 min using (BimC4A)_3_-Ascorbate stock solution (5 μL, see S1 File) as the catalyst. ^89^Zr-Cy5.5-Folate-FH (^**89**^**Zr-12**) was purified, collected, and concentrated as for ^89^Zr-Cy5.5-Azide-FH (^**89**^**Zr-8**). RCY: 0.119 mCi, 68.8%; Specific activity: 33.3 mCi/mmol Fe; RCP >99% (S6 Fig).

### Synthesis of RGD functionalized FHs, (RGD-FH, 14), see Fig 4

Azide-FH (**4,** 0.2 mg Fe) and DBCO-PEG_4_-RGD (**13**, 5 μL, 15.6 mM in DMSO) was incubated under room temperature in chelexed water (200 μL) for 1 hr. The mixture was purified by PD-10 column (PBS) and concentrated by ultrafiltration. A ratio of RGD/FHNP (11.7, Table 1) was obtained by reaction with DBCO-Cy5.5 (**6)**.

### Synthesis of protamine functionalized FHs, (Cy5.5-Protamine-FH, 16 and ^89^Zr-Cy5.5-Protamine-FH, ^89^Zr-16), see Fig 4

*For Cy5*.*5-Protamine-FH* ***(16)*,** to a solution of Azide-FH (**4,** 0.2 mg Fe, 40 μL) and DPBS (160 μL), was added DBCO-Protamine-Cy5.5 (**15**, 15 μL, 0.12 mM in chelexed water) under stirring. (Synthesis of DBCO-Protamine-Cy5.5 (**15**) is given in S2 File). The mixture was incubated (room temperature, 30 min). The mixture was loaded on a PBS preconditioned Sephadex G-50 column, eluted with PBS and concentrated by ultrafiltration. A ratio of Protamine/FHNP (3.4, Table 1) was obtained by UV absorbance of Cy5.5. The attachment of protamine was confirmed by a fluorescence increase after the trypsin digestion (S2 File), which cleaves the high arginine protamine and reduces fluorescence quenching.

*For* ^*89*^*Zr-Cy5*.*5-Protamine-FH (*^***89***^***Zr-16****)*, *to a* 0.3 mL glass reactivial with ^89^Zr-Azide-FH (^**89**^**Zr-4**) (0.5 mg Fe, 0.457 mCi) in DPBS (0.25 mL), was added the stock solution of DBCO-protamine-Cy5.5 (**15**, 25 μL, 0.12 mM in chelexed water). In order to get a more homogeneous payload distribution, the stock solution of DBCO-Protamine-Cy5.5 (**15**) was added under well stirring by fractional additions for 5 times with 5 μL every 3 min. After the complete addition of DBCO-Protamine-Cy5.5 (**15**), the mixture was further stirred under room temperature for 30 min. A DPBS conditioned Sephadex G-50 column was employed for purification. The product was collected and reconstituted in DPBS (1.4 mL) without centrifugation. The iron concentration (6.66 mM) and protamine/NP ratio (2.6, Table 1) were measured with UV absorbance. RCY: 0.387 mCi, 84.7%. RCP was analyzed by PD-10 SEC analysis (RCP > 99%, **see** S7 Fig). Specific activity: 43.2 mCi/mmol Fe.

### Cell binding study of RGD-FH (14) with BT-20 cells (Fig 5)

**Fig 5:**
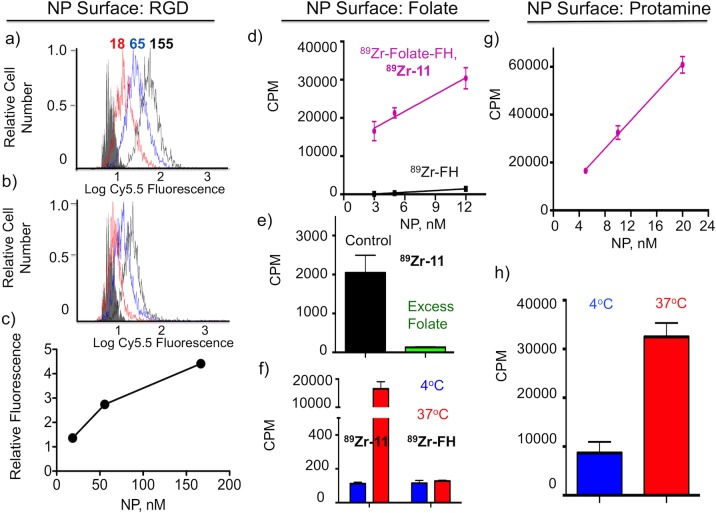
Bioactivity of RGD, Folate and Protamine functional groups attached through click reactions to the FH NP surface. *(a)* Flow cytometry histograms for the reaction of RGD-FH (**14)** and a control NP (b) at three NP concentrations (18 nM, 65 nM, and 155 nM) with BT-20 cells are shown. NP concentrations were determined from iron concentrations using the manufacture’s value of 5874 Fe’s per NP (AMAG Pharmaceuticals Package insert.) Relative fluorescence (c) from (a) and (b) versus NP concentration is shown. Relative fluorescence is the mean fluorescence of (a) minus the mean fluorescence of (b) divided by the fluorescence of unstained cells. (d) Uptake of the ^89^Zr-Folate-NP **(**^**89**^**Zr-11)** and a ^89^Zr-FH control NP is shown in at three NP concentrations. (e) Uptake was blocked by 1 μM folate (**10)** and was highly temperature dependent (f). (g) Uptake of ^89^Zr-Cy5.5-Protamine-NP (^89^**Zr-16**) at three different NP concentrations is shown. (h) ^89^Zr-Cy5.5-Protamine-FH (^**89**^**Zr-16**) uptake at 4°C and 37°C is shown (10 nM NPs were used). MDA-MB-231 cells were used for (d) through (h).

(125,000 cells/well) were plated in a 12-well plate on day 0. On day 1, media was aspirated and cells were washed with FBS-augmented DPBS (2% FBS). Cells were then incubated with 167 nM, 55.5 nM, or 18.5 nM nanoparticle suspended in DPBS (2% FBS). After 0.5 h, media was aspirated, cells were washed twice with DPBS (2% FBS), trypsinized and resuspended in DPBS, for measurement by single channel flow cytometry (BD-LSRII Cytometer).

### Cell binding study of ^89^Zr-Folate-FH (^89^Zr-11) with MDA-MB-231 cells (Fig 5d–5f)

Cells were grown according to the supplier’s recommendation in a humidified incubator at 37°C, 5% CO_2_. Two hundred thousand cells were seeded on 6-well Costar plates (Corning, NY) in a 2 mL media, followed by overnight incubationat 37°C, 5% CO_2_. They were then treated with 1X PBS-diluted compound, and placed in the incubator for 6 h. Cells were washed three times with 1X PBS at 37°C, followed by a 10 min lysis in RIPA buffer. The solution was then transferred to cytometry tubes, and radioactivity was measured (Perkin Elmer Wizard 2480 Gamma Counter). To block endocytic uptakeof 89Zr-Folate-FH, the cells were incubated for1 h at 4°C with the NPs (final concentration = 3 nM). Control cells were incubated with HIR labeled FH,^89^Zr-FH NP (final concentration = 10 nM) for 2 h (37°C or 4°C). Alternatively, ^89^Zr-Folate-FH NPs (final concentration = 1 nM) were incubated with cells and folate ((**10**) @ 1 μM) for 1 h at 37°C.

### Cell binding study of ^89^Zr-Cy5.5-Protamine-FH (^89^Zr-16) with MDA-MB-231 cells(Fig 5g and 5h)

For the uptake of the radiolabeled Protamine-FH compound, two hundred thousand cells were seeded on 6-well Costar plates (Corning, NY) in 2 mL media, followed by overnight incubation at 37°C, 5% CO2. They were then treated with 1X PBS-diluted compound, and placed in the incubator for 2 h. Subsequently, they were washed three times with 1X PBS, which was warmed at 37°C, followed by 10-minute lysis in the presence of RIPA buffer. Prevention of endocytic uptake was performed by placing the cells for 1 h at 4°C in the presence of the nanoparticles (final concentration = 10 nM). Cell-associated radioactivity was performed similar to the study investigating the uptake of radiolabeled-folate-FH.

## Results

### Syntheses of azide and alkyne functionalized FH NPs

The carboxyl groups of carboxymethyl dextran (CMD) create a heat resistant bond between the iron oxide [11, 12] and provide sites for reaction with amines [13, 14]. So FH (**1**) was reacted with Azido-PEG_3_-Amine (**2**) or Alkyne-PEG_4_-Amine (**3**) using HOBT and EDC as shown in Fig 2a.

To demonstrate the presence of click reactive groups on the putative Azide-FH (**4**) or Alkyne-FH (**5**), NPs were reacted click reactive fluorochromes, DBCO-Cy5.5 (**6**) or Azide-Cy5.5 (**7**). (Structures are given in the Supplement, S8 Fig.) After size exclusion chromatography to remove unreacted fluorochrome, the concentration of Cy5.5 was determined spectrophotometrically (absorbance @ 675 nm, extinction coefficient = 250,000 M^-1^ cm^-1^), see Fig 2b and 2c. With the iron concentration determined and using the manufacturer’s figure of 5874 Fe per NP, there were 14.9 reactive azides based on the value 14.9 Cy5.5s/NP reactive per NP (Fig 2b). Similarly, there were 13.1 reactive alkynes per NP (Fig 2c). Values are summarized in Table 1.

To demonstrate that the association between Cy5.5 and NPs resulted from a click-based covalent reaction, and not adsorption of Cy5.5 to the NP surface two types of controls were run. First, the reaction between Azide-FH (**4**) and DBCO-Cy5.5 (**6**) was determined after incubation with DBCO-NH_2_ which reduced n from 14.3 to 1.3 (Fig 2d). Second, DBCO-Cy5.5 (**6**) was reacted with FH (**1**) and gave no detectable reaction **(**Fig 2e). As summarized in Table 1, FH can be derivatized with click reactive azide or alkyne groups and the numbers of reactive groups per NP determined by reaction with an appropriate Cy5.5 fluorochrome.

### ^89^Zr HIR of Azide-FH (4) and Alkyne-FH (5) and azide and alkyne’s tolerance of HIR proved by the reaction with the “clickable” Cy5.5 fluorochromes (which are compounds DBCO-Cy5.5 (6) and Azide-Cy5.5 (7) in Fig 2.)

The Azide-FH (**4**) and Alkyne-FH (**5**) were radiolabeled with ^89^Zr by HIR, yielding ^89^Zr-Azide-FH (^**89**^**Zr-4**) and ^89^Zr-Alkyne-FH (^**89**^**Zr-5**), which were then reacted with click reactive Cy5.5’s, see Fig 2a with results summarized in Table 1. (Sizes and relaxivities were determined only for non-radioactive NPs to avoid possible instrument contamination.) The reaction of ^89^Zr-Azide-FH (^**89**^**Zr-4**) and ^89^Zr-Alkyne-FH (^**89**^**Zr-5**) with Cy5.5 indicates the numbers of reactive groups per NP and provides a path radioactive, magnetic and fluorescent multimodal NPs for future use. Results summarized in Table 1 indicate FH can be functionalized with alkyne or azide that survive HIR. The reaction of ^89^Zr-Azide-FH (^**89**^**Zr-4**) or ^89^Zr-Alkyne-FH (^**89**^**Zr-5**) (or their non-radioactive counterparts) with targeting groups was therefore undertaken.

### Click conjugation of biomolecule targeting groups on HIR and non-HIR FH NPs

To demonstrate the use of the HIR and click chemistry surface modification combination for the design of targeted NPs, we selected three well-studied targeting groups (folate, an RGD peptide, protamine) for study. The interaction of folate-bearing conjugates with the folate receptor has been used to target NIR fluorochromes [15, 16], radioisotopes [17–19], polymers [20] and NPs [21–23]. Similarly, the interaction of RGD peptides with RGD binding integrins [24–27] has been used to target a wide range of radioisotopes [28, 29], fluorochromes [30, 31], polymers [32], and NPs [30, 33]. RGD binding integrins are upregulated on angiogenic endothelial cells. Finally, high arginine peptides like protamine serve as a membrane translocation signals, inducing internalization of nucleic acids and NPs [34–37].

#### Folate attachment by a copper mediated click reaction

We employed a copper mediated click reaction between the alkyne-functionalized FH’s (^89^Zr-Alkyne-FH, ^**89**^**Zr-5** or Alkyne-FH, **5**) and Azide-Folate (**10**)**,** to obtain folate functionalized NPs (^89^Zr-Folate-FH, ^**89**^**Zr-11** or Folate-FH, **11**) as shown in Fig 3. To demonstrate the presence of folates on the NP surface, NPs were reacted with an azide-bearing Cy5.5 (**7)** before and after their reaction with azide-folate. The number of folates per NP, p, was then determined by subtraction, p = m—q, where m is the number of reactive alkynes before folate attachment and q is the number of reactive alkynes remaining after folate attachment (Table 1). Using this technique there were 12.2 reactive alkynes on ^89^Zr-Alkyne-FH (^**89**^**Zr-5**) and 12.2 folates on ^89^Zr-Cy5.5-Alkyne-FH (^**89**^**Zr-9**). We term this as the*“Before and After Fluorochrome Reaction and Subtraction Method”* for determination of targeting groups on NP surfaces.

#### RGD and protamine attachment by copperless click reactions

To demonstrate a surface modification of ^89^Zr-Azide-FHs (^**89**^**Zr-4**) or Azide-FH (**4**) using a copperless click reaction, the NP was reacted with a DBCO-bearing RGD peptide (Fig 4), and the number of surface RGDs determined with the *“Before and After Fluorochrome Reaction and Subtraction Method”* to be 11.7 RGD’s per NP.

To obtain a protamine functionalized NPs, a protamine with an N-terminal DBCO and C-terminal Cy5.5 was synthesized. (Synthesis of DBCO-Protamine-Cy5.5 (**15**) is provided in S2 File). Unlike folate or RGD targeting molecules, only small numbers of protamines per NP could be attached without agglomeration (Fig 4). Hence the numbers of protamines per NP (2.6–3.4) was demonstrated directly from the numbers of Cy5.5s on NPs (Cy5.5-Protamine-FH, **16** or ^89^Zr-Cy5.5-Protamine-FH, ^**89**^**Zr-16**) as summarized in Table 1.

### Bioactivity of surface functionalized NPs

To demonstrate that the surface functionalized NPs synthesized possess bioactive functional groups three experiments were conducted (Fig 5): (i) the binding of RGD functionalized NPs (RGD-FH, **14**, Fig 4) on integrin expressing cells was examined using fluorescence (flow cytometry, Fig 5**,** Panel of RGD NP). (ii) the binding of HIR labeled, folate functionalized NPs **(**^89^Zr-Folate-FH, ^**89**^**Zr-11**, Fig 3**)** using folate receptor bearing cells was examined (Fig 5**,** Panel of Folate NP**)**. (iii) The uptake of HIR labeled protamine functionalized NPs (^89^Zr-Cy5.5-Protamine-FH**,** ^**89**^**Zr-16**, Fig 4) was examined (Fig 5**,** Panel of Protamine NP**)**.

#### RGD-Cy5.5-FH bioactivity

A NP Cy5.5-Azide-FH (**8**) bearing 8.7 Cy5.5/NP s and second bearing 8.7 Cy5.5/NP and 18.8 RGDs/NP (**RGD-Cy5.5-FH**) were synthesized by reacting Azide-FH (**4**) with DBCO-Cy5.5 (**6**)**,** or by reacting Cy5.5-Azide-FH (**8**) with DBCO-PEG_4_-RGD **(13).** The above fluorescent version of RGD bearing NP (**RGD-Cy5.5-FH**) and control NPs Cy5.5-Azide-FH (**8**) were incubated with BT-20 integrin-expressing cells [38, 39] and cell fluorescence examined by flow cytometry (Fig 5a and 5b). Fluorescence was higher for the RGD bearing RGD-FH (**14**) than the control NP at three NP concentrations, indicating NPs-bearing RGD peptides bound to cellular integrins (Fig 5c). The RGD peptides on RGD-FH (**14**) bind integrins expressed on BT-20 cells.

#### ^89^Zr-Folate-FH bioactivity

The interaction of ^89^Zr-Folate-FH (^**89**^**Zr-11**) (Fig 3, Folate/NP = 9.0) with folate receptor-bearing MDA-MA-231 cells [8, 40] was examined as shown Fig 5d–5f with HIR ^89^Zr-FH NP [5] as a control for nonspecific binding. NPs were used at three concentrations (Fig 5d), with far higher uptake by folate bearing ^89^Zr-Folate-FH (^**89**^**Zr-11**) than the non-folate bearing control, ^**89**^**Zr-FH.** In addition, folate **(10)** at 1 μM blocked more than 90% of the uptake of ^89^Zr-Folate-FH (^**89**^**Zr-11,** Fig 5e). ^89^Zr-Folate-FH (^**89**^**Zr-11**) uptake was temperature dependent, indicating uptake was by an energy requiring process like endocytosis (Fig 5f). On the other hand, uptake of ^**89**^**Zr-FH** was far lower and temperature independent. Folate groups on ^89^Zr-Folate-FH (^**89**^**Zr-11**) are bioactive, binding folate receptors on MDA-MB-231 cells.

#### ^89^Zr-Cy5.5-Protamine-FH (^89^Zr-16) bioactivity

To examine the membrane translocating bioactivity of the arginine-rich protamine peptide on the NP surface, we incubated ^89^Zr-Cy5.5-Protamine-FH (^**89**^**Zr-16**) with MDA-MB-231 cells at three concentrations (Fig 5g**)**. This translocating bioactivity was greatly reduced at (4°C, Fig 5h). Protamine on ^89^Zr-Cy5.5-Protamine-FH (^**89**^**Zr-16**) is bioactive, inducing an uptake of ^89^Zr-Cy5.5-Protamine-FH (^**89**^**Zr-16**) (Fig 5h) than that of ^**89**^**Zr-FH** shown in **(**Fig 5f**).**

## Discussion

Our scheme of Fig 1 provides fluorescent and/or ^89^Zr labeled NPs (Cy5.5-Azide-FH (**8**), ^89^Zr- Cy5.5-Azizde-FH (^**89**^**Zr-8**)**,** Cy5.5-Alkyne-FH (**9**) and ^89^Zr-Cy5.5-Alkyne-FH (^**89**^**Zr-9**)) that are internalized by the phagocytic monocyte/macrophage system, an internalization that can occur either in vivo or ex-vivo [5]. **Scheme 1** also provides a chemistry for the in vitro loading of non-phagocytic cells such as T lymphocytes, which do not internalize magnetic nanoparticles unless a translocation signal is involved [35, 37, 41]. Specifically, protamine can be attached to the FH NP that is internalized by mesenchymal stem cells and U87 cells [42]. Thus some NPs described here will be internalized by the monocyte arm of the immune system while others (e.g. the protamine constructs ^89^Zr-Cy5.5-Protamine-FH (^**89**^**Zr-16**) or Cy5.5-Protamine-FH (**16**)) will be internalized by its lymphocyte arm. The NPs described here can be used for the ex-vivo labeling, and subsequent tracking of the various arms of the immune system.

We have shown that both azides and alkynes can be attached to FH and withstand HIR, allowing a wide range of click chemistry surface functionalizations with radioactive or non-radioactive NPs. However, with iron oxide NPs like FH the reducing conditions used for some click reactions can reduce ferric to ferrous ions, a reaction evident by a change from a reddish to a black/brown color. Our conditions for copper mediated click reactions of the Alkyne-FH (**5)** with Azide-Cy5.5 **(7)** or Azide-Folate (**10)** were 0.2 mg Fe, 15 min, room temperature with molar ratios of 67 / 1 / 1 / 37 for Fe / Cu / (BimC_4_A)_3_ / Na.Ascorbate in DPBS. In addition the copper catalyst should be freshly made and solutions should be oxygen free (see S1 File). As shown in Table 1, the measurements of nanoparticle size and relaxivity indicate a lack dissolution or aggregation due to surface functionalization. Copperless click reactions between Azide-FH (**4**) and click reactive, strained cyclic DBCO were used with RGD and protamine peptides; these are free from the issues than arise with the use of reductive conditions.

To determine the numbers of chemically reactive azide or chemically reactive alkyne functional groups on the NP surface we employed the “*Before and After Fluorochrome Reaction and Subtraction Method*.” This was done because of the shortcomings of other analytical methods discussed below, with Cy5.5 selected as an optical reporter because of its high extinction coefficient, its lack of spectral overlap with iron oxides (see Fig 2b), and the commercial availability of click reactive Cy5.5s. Shortcomings of other analytical arise when engaging in defining nanoparticle chemistry for nanoparticles used in mouse imaging. This arises because typically small quantities of nanoparticles are synthesized and used, since one milligram is sufficient to inject four 25 g mice at a typical dose of 10 mg Fe/kg. Hence analytical methods for this setting must consume small amounts of NPs, as well as overcoming NP superparamagnetism (no NMR) and the intense UV and visible absorption of iron oxides. Here we show how reactions with a commercially available fluorochrome can be used to obtain the number of biomolecules per NP. By reacting commercially available, click reactive Cy5.5s with NPs, the number attached Cy5.5s can be determined spectrophotometrically, with the number of bioactive groups, being the difference between the number of Cy5.5s attached before and number attached after their reaction the biomolecule. (Fig 3). With the high extinction coefficient of Cy5.5, the reaction of Cy5.5 with NPs uses only about 0.02 mg Fe to interrogate the status of the NP surface. Conventional methods of analyzing NP surfaces, e.g. FTIR or SIMMS [43] require substantially greater NP amounts and specialized instrumentation. With this “Before and After Fluorochrome Reaction and Subtraction Method” the numbers of folates or RDGs were determined through the reaction of Cy5.5. The method is useful when the number of attached targeting groups per NP is high, and difference of the before after reaction (p = m- q, Fig 3) is large. However, to determine low numbers protamines attached per NP, (a low number being necessitated by protamine induced NP agglomeration, see below), we synthesized a DBCO-protamine-Cy5.5 (**15**, Fig 4**,** see S2 File), and determined the numbers of protamines per NP directly.

With regard to steric blockage with Cy5.5 dye attachment to the Feraheme nanoparticle surface, steric blockage is not seen because of the Feraheme nanoparticle’s extremely large dimensions compared to a Cy5.5. The Feraheme nanoparticle has diameter of 25 nm, equivalent to a globular protein of 750 kDa [44]. The Cy5.5 dyes have molecular weights of about 700–800 daltons, so that attachment of, for example 20 Cy5.5s (MW = 16,000 Da), is readily accommodated on the Feraheme nanoparticle. Thus we have not observed steric blocking and the number of Cy5.5s can be used to represent the reactive sites under click chemistry surface modification employed in this study.

A consideration when performing NP surface chemistry on FH NP is avoiding NP agglomeration. FH (**1**) has a strong negative charge over a wide pH range, with a Zeta potential at pH 7.36 of -30.55 mV [9]. With our chemistry, FH (**1**) tolerated the attachment of negatively charged Cy5.5, the cRGD peptide or folate at levels of 10–20 targeting groups per NP with a maintenance of NP size of less than 50 nm, see Table 1. However, when highly positively charged protamine was employed, agglomeration was initially obtained. Protamine 1 consists of 32 amino acids of 22 of which are positive arginines and the rest are neutral amino acids [35]. (Protamine is a mixture of 4 similar peptides.) To attach the smaller numbers of protamines consistent with NP stability, and determine the number of protamines per NP, we synthesized a protamine with N-terminal DBCO and C-terminal Cy5.5 (DBCO-protamine-Cy5.5 (**15**)**,** see S2 File), and determined the number of protamines per NP directly from the attachment of DBCO-Protamine-Cy5.5 (**15**)**,** see Fig 4.

Our surface modifications with Cy5.5 fluorochromes, folate, RGD peptide and protamine employed materials with molecular weights of less than 5000 daltons. Separation of radioactive nanoparticles from these low molecular weight, surface functionalizing compounds is readily accomplished with PD-10 and Sephadex G-50 columns. With our method, a wide range of nanoparticle surface functionalization strategies can be employed with similarly sized materials. However, use of larger biomolecules like antibodies will required a different separation method and may occur far more slowly under the conditions used here.

The combination of heat-induced NP radiolabeling and click chemistry NP surface functionalization outlined in Fig 1 provides a number of positive features. *First*, it employs Feraheme NPs as a starting NP, which is an approved drug. FH NPs have well-described physical properties,[9, 44, 45] a well-understood metabolism,[46–48] low toxicity,[49, 50] low batch to batch variability, and the sterility required of a parenteral pharmaceutical. Due to the superparamagnetism of its iron oxide core, FH (**1**) has been widely used as an MR contrast agent,[51] which further aids in understanding diagnostic applications of this NP. *Second*, the carboxyl groups of FH’s carboxymethyldextran (CMD) coating endow the NP with extraordinary heat stability[11, 12, 52] and can be reacted with primary amines.[13, 42] CMD’s carboxyl groups are not involved in the radiocation binding of the HIR reaction based on electron magnetic resonance indicating their binding to the iron oxide surface and the ability to perform HIR with NP’s lacking carboxyl groups. [4] Thus, we have modified some of these carboxyl groups to obtain alkyne and azide functionalized NPs as shown in Fig 2. *Third*, FH’s heat stability allows heat-induced radiolabeling, which permits the bonding of three cations used in nuclear imaging (^111^In, ^89^Zr, ^64^Cu) without altering the NPs physical properties or pharmacokinetics.[4, 5] Here we demonstrate a fourth advantage of FH NPs, that alkyne and azide functionalized FH NPs (Azide-FH, **4** & Alkyne-FH**, 5**) survive the HIR reaction (yielding ^89^Zr-Azide-FH, ^**89**^**Zr-4** & ^89^Zr-Alkyne-FH, ^**89**^**Zr-5**). This allows syntheses of nonradioactive Azide-FH (**4**) and Alkyne-FH (**5**) at modest pace and desired scale, followed by HIR, followed by the rapid click chemistry attachment of biomolecules under mild reaction conditions (Fig 3). Here Azide-FH (**4**) or Alkyne-FH (**5**) were radiolabeled, surface modified, and purified in total time of less than 4 h, which is short compared to the radiochemical half-lives of ^64^Cu, ^111^In or ^89^Zr.

## Conclusions

As summarized in Table 1, the HIR/click combination is a powerful approach for generating multifunctional nanomaterials, which allows detection of the NPs made here by MRI (FH’s superparamagnetism), by fluorescence (click attachment of fluorochromes), and by radioactivity (HIR). In the future, the general strategy outlined in Fig 1 may be used to obtain multimodal NPs for labeling circulating monocytes or resident macrophages,[5] for targeting NPs to cells expressing receptors (e.g. folate receptor, RGD binding integrins) or for the ex-vivo labeling cells through the membrane translocating activity of protamine.[35, 37, 41] The reactions demonstrated here, use of the widely available and well understood FH (**1**) for the synthesis of the nonradioactive Alkyne-FHs (**5**) and Azide-FH (**4**) intermediates, followed by HIR, then by click chemistry modification of the NP surface biomolecules, provides a path to the design of multimodal/multifunctional NPs suitable for a wide range of diagnostic and therapeutic applications.

## Supporting information

S1 Fig RCP analysis for ^89^Zr-Azide-FH (^89^Zr-4). (DOCX)

S2 Fig RCP analysis of ^89^Zr-Alkyne-FH (^89^Zr-5). (DOCX)

S3 Fig RCP analysis ^89^Zr-Cy5.5-Azide-FH (^89^Zr-8). (DOCX)

S4 Fig RCP analysis ^89^Zr-Cy5.5-Alkyne-FH (^89^Zr-9). (DOCX)

S5 Fig RCP analysis ^89^Zr-Folate-FH (^89^Zr-11). (DOCX)

S6 Fig RCP analysis ^89^Zr-Cy5.5-Folate-FH (^89^Zr-12). (DOCX)

S7 Fig RCP analysis ^89^Zr-Cy5.5-Protamine-FH (^89^Zr-16). (DOCX)

S8 Fig Structures of RGD-DBCO, Folate-azide, Cy5.5-DBCO, Cy5.5-azide. (DOCX)

S1 File Preparation of the stock solution for copper click catalyst. (DOCX)

S2 File Preparation of DBCO-Protamine-Cy5.5 (15) in Fig 4. (DOCX)

## Abbreviations

(BimC_4_A)_3_: tripotassium 5,5′,5′′-[2,2′,2′′-nitrilotris(methylene)tris(1*H*-benzimidazole-2,1-diyl)]tripentanoate hydrate)
^89^Zr-Cy5.5-Alkyne-FH: ^89^Zr-labeled Cy5.5 fluorescent FH NPs through Alkyne-FH copper catalyzed click chemistry
^89^Zr-Cy5.5-Azide-FH: ^89^Zr-labeled Cy5.5 fluorescent FH NPs through Azide-FH copperless click chemistry
^89^Zr-Cy5.5-Protamine-FH: ^89^Zr-labeled Protamine modified FH NPs
^89^Zr-Folate-FH: ^89^Zr-labeled folate modified FH NPs
^89^Zr-RGD-FH: ^89^Zr-labeled cRGD peptide modified FH NPs
Alkyne-FH: alkyne modified FH NPs
Azide-FH: azide modified FH NPs
CMD: carboxymethyl dextran
Cy5.5-Alkyne-FH: Cy5.5 fluorescent FH NPs through Alkyne-FH copper catalyzed click chemistry
Cy5.5-Azide-FH: Cy5.5 fluorescent FH NPs through Azide-FH copperless click chemistry
Cy5.5: cyanine 5.5 fluorochrome
Cy5.5-Protamine-FH: Protamine modified FH NPs
DBCO: dibenzocyclooctyne
DFO: deferoxamine
DMSO: dimethyl sulfoxide
DPBS: Dulbecco’s phosphate buffered saline without calcium and magnesium
EDC: 1-ethyl-3-(3-dimethylaminopropyl)carbodiimide
FBS: fetal bovine serum
FH: Feraheme
Folate-FH: folate modified FH NPs
HIR: Heat-Induced Radiolabeling
HOBT: 1-hydroxybenzotriazole
ICP-MS: inductively coupled plasma mass spectrometry
MES: 2-(N-morpholino)ethanesulfonic acid
MRI: magnetic resonance imaging
NPs: nanoparticles
PD-10: desalting column with Sephadex G-25 resin
PET: positron emission tomography
RCP: radiochemical purity
RCY: radiochemical yield
RGD-FH: cRGD peptide modified FH NPs
SPECT: single photon emission computed tomography
TFA: trifluoroacetic acid
