# Supplementary material for: Heat-induced-radiolabeling and click chemistry: A powerful combination for generating multifunctional nanomaterials
Source: PLoS One. 2017 Feb 22;12(2):e0172722. doi: 10.1371/journal.pone.0172722 (PMC5321420; doi:10.1371/journal.pone.0172722)
Supplement: S1 Fig — (DOCX) [file pone.0172722.s001.docx]

*^89^Zr-Azide-FH (****^89^Zr-4****,* ***Fig 2****)* was analyzed by PD-10 column (**S1 Fig** below).

**S1 Fig. RCP analysis for ^89^Zr-Azide-FH (^89^Zr-4)** by PD-10 gel filtration eluted by PBS
