# Supplementary material for: Heat-induced-radiolabeling and click chemistry: A powerful combination for generating multifunctional nanomaterials
Source: PLoS One. 2017 Feb 22;12(2):e0172722. doi: 10.1371/journal.pone.0172722 (PMC5321420; doi:10.1371/journal.pone.0172722)
Supplement: S2 Fig — (DOCX) [file pone.0172722.s002.docx]

*^89^Zr-Alkyne-FH (****^89^Zr-5,*** ***Fig 2****)* was analyzed with a PD-10 column (**S2 Fig** below).

**S2 Fig. RCP analysis of ^89^Zr-Alkyne-FH (^89^Zr-5)** by PD-10 gel filtration eluted by PBS
