# Supplementary material for: Heat-induced-radiolabeling and click chemistry: A powerful combination for generating multifunctional nanomaterials
Source: PLoS One. 2017 Feb 22;12(2):e0172722. doi: 10.1371/journal.pone.0172722 (PMC5321420; doi:10.1371/journal.pone.0172722)
Supplement: S3 Fig — (DOCX) [file pone.0172722.s003.docx]

*^89^Zr-Cy5.5-Azide-FH* ***(^89^Zr-8,*** ***Fig 2****)* was analyzed by a PD-10 column (**S3 Fig**).

| **S3 Fig. RCP analysis ^89^Zr-Cy5.5-Azide-FH (^89^Zr-8)** by PD-10 gel filtration eluted by PBS |  |
| --- | --- |
