# Supplementary material for: Heat-induced-radiolabeling and click chemistry: A powerful combination for generating multifunctional nanomaterials
Source: PLoS One. 2017 Feb 22;12(2):e0172722. doi: 10.1371/journal.pone.0172722 (PMC5321420; doi:10.1371/journal.pone.0172722)
Supplement: S5 Fig — (DOCX) [file pone.0172722.s005.docx]

*^89^Zr-Folate-FH* ***(^89^Zr-11, Fig 3****)* was analyzed by a PD-10 column (**S5 Fig**).

**S5 Fig. RCP analysis ^89^Zr-Folate-FH (^89^Zr-11)** by PD-10 gel filtration eluted by PBS
