# Supplementary material for: Heat-induced-radiolabeling and click chemistry: A powerful combination for generating multifunctional nanomaterials
Source: PLoS One. 2017 Feb 22;12(2):e0172722. doi: 10.1371/journal.pone.0172722 (PMC5321420; doi:10.1371/journal.pone.0172722)
Supplement: S7 Fig — (DOCX) [file pone.0172722.s007.docx]

*^89^Zr-Cy5.5-Protamine-FH (****^89^Zr-16, Fig 4****)* was analyzed by a PD-10 column (**S8 Fig**).

**S8 Fig. RCP analysis ^89^Zr-Cy5.5-Protamine-FH (^89^Zr-16)** by PD-10 gel filtration eluted by PBS
