# Supplementary material for: Heat-induced-radiolabeling and click chemistry: A powerful combination for generating multifunctional nanomaterials
Source: PLoS One. 2017 Feb 22;12(2):e0172722. doi: 10.1371/journal.pone.0172722 (PMC5321420; doi:10.1371/journal.pone.0172722)
Supplement: S8 Fig — (DOCX) [file pone.0172722.s008.docx]

**S9 Fig.** **Structures of RGD-DBCO, Folate-azide, Cy5.5-DBCO, Cy5.5-azide**
