# Supplementary material for: Heat-induced-radiolabeling and click chemistry: A powerful combination for generating multifunctional nanomaterials
Source: PLoS One. 2017 Feb 22;12(2):e0172722. doi: 10.1371/journal.pone.0172722 (PMC5321420; doi:10.1371/journal.pone.0172722)
Supplement: S1 File — (DOCX) [file pone.0172722.s009.docx]

**Preparation of the stock solution for copper click catalyst**

A stock solution of copper click catalyst was prepared by dissolving the following compounds in a order of: CuSO_4_ (8.85mg, 0.055mmol) in deoxygenated PBS (5ml), (BimC4A)_3_ (44.5mg, 0.054mmol), and sodium L-ascorbate (397.6mg, 2 mmol).
