# Supplementary material for: Heat-induced-radiolabeling and click chemistry: A powerful combination for generating multifunctional nanomaterials
Source: PLoS One. 2017 Feb 22;12(2):e0172722. doi: 10.1371/journal.pone.0172722 (PMC5321420; doi:10.1371/journal.pone.0172722)
Supplement: S2 File — (DOCX) [file pone.0172722.s010.docx]

**Synthesis of DBCO-Protamine-Cy5.5 (15).**

Preparation of this peptide was according the scheme below.

**Scheme** **Preparation of DBCO-Protamine-Cy5.5 (15) in Fig 4**

*Synthesis of DBCO-Protamine*

To attach DBCO to the N-terminus of protamine, protamine (2ml, 5 μmol, 10mg/ml in 0.9% saline) in PBS (pH 7.4, 0.4ml) was incubated with DBCO-PEG4-NHS ester (2eq, 10 μmol) in DMSO (0.4ml) at 4^o^C for overnight. Purification was by C18 HPLC with a gradient from 0% B to 100%B in 20min, then back to 0% B in 2min and isocratic for 3min; flow: 15ml/min; 225nm; column: Proto 300 C18 10μm, 250x20mm (Higgins Analytical Inc., P/N: RS-2520-W181). Yield: 10.7mg, 45%. The product (DBCO-Protamine) was a white powder and characterized by MALDI-TOF and ESI MS (**Table).**

*Synthesis of DBCO-Protamine-Cy5.5 (****15****)*

To react the C-terminus of DBCO-Protamine with Cy5.5, a mixture of DBCO-Protamine (9.6 mg, 2.0 μmol), Cy5.5-amine (4.5 mg, 6.0 μmol), EDC (12.7mg, 66 μmol), DIPEA (22.6 μl, 0.13 mmol), and HOBT (3.3 mg, 21.6 μmol) in DMF (1 ml), was incubated at room temperature for 48 hr. HPLC purification employed a gradient from 30% B to 100%B in 20min, then back to 30% B in 5min and isocratic for 5min; flow: 4ml/min; 225nm; column: Targa C18 5μm, 250x10mm(Higgins Analytical Inc., P/N: TS-2510-C185). Yield: 25%. The product (DBCO-Protamine-Cy5.5, **15**), a blue powder, was characterized by MALDI-TOF and ESI MS (**Table)**

**Table** **MS (m/z) data of Protamine, DBCO-Protamine, and DBCO-Protamine-Cy5.5** (**15**)

| **Compound** | **MALDI** | | **ESI** | | |
| --- | --- | --- | --- | --- | --- |
| **Protamine** | Obs. | 4068, 4254, 4323  [M+H]^+^ | 813, 850, 864  [M+5H]^5+^ | 677.7, 708.7, 720.2  [M+6H]^6+^ |  |
|  | Cal. |  | 4061, 4246, 4316  [M+H]^+^ |  |  |
| **DBCO-Protamine** | Obs. | 4646, 4832, 4901  [M+H]^+^ | 1160.5, 1207.0, 1224.3  [M+4H]^4+^ | 928.5, 965.8, 979.6  [M+5H]^5+^ | 774.0, 804.8, 816.5  [M+6H]^6+^ |
|  | Cal. | 4650, 4837, 4906  [M+H]^+^ | 4639, 4824, 4894  [M+H]^+^ | | |
| **DBCO-Protamine-Cy5.5 (15)** | Obs. | 5311, 5497, 5566  [M^+^] | 1061, 1098, 1112.5  [M+4H]^5+^ | 758.5, 784.9, 795  [M+6H]^7+^ | 663.8, 686.8, 695.5  [M+7H]^8+^ |
|  | Cal. | 5312, 5495, 5564,  [M^+^] | 5302, 5487, 5557  [M^+^] | | |

*Trypsin digestion of Cy5.5-Protamine-FH* ***(16, Fig 4)***

The fluorescence increase when Cy5.5-Protamine-FH (**16)** is subjected to trypsin digestion in shown in **Fig** below. See Kircher MF et al (2004) “[A dual fluorochrome probe for imaging proteases.](http://www.ncbi.nlm.nih.gov/pubmed/15025519)” Bioconjug Chem. 15(2):242-8; Kircher MF, et al (2002) “[Ratio imaging of enzyme activity using dual wavelength optical reporters.](http://www.ncbi.nlm.nih.gov/pubmed/12920849)” Mol Imaging. 1(2):89-95.

**Fig.** **Time course of fluorescence increase with trypsin digestion of Cy5.5-Protamine-FH (16).** The data points represent differences of the fluorescence intensity at 697nm
